# A privacy-preserving distributed filtering framework for NLP artifacts

**DOI:** 10.1186/s12911-019-0867-z

**Published:** 2019-09-07

**Authors:** Md Nazmus Sadat, Md Momin Al Aziz, Noman Mohammed, Serguei Pakhomov, Hongfang Liu, Xiaoqian Jiang

**Affiliations:** 1 Department of Computer Science, University of Manitoba, Winnipeg, MB R3T 2N2 Canada; 2 Department of Biomedical Informatics, University of California San Diego, La Jolla, CA USA; 3 Department of Pharmaceutical Care & Health Systems, University of Minnesota, Minneapolis, MN USA; 4 Department of Health Sciences Research, Mayo Clinic College of Medicine, Rochester, MN USA; 5 School of Biomedical Informatics, University of Texas Health Science Center at Houston, Houston, TX USA

**Keywords:** Biomedical data security and privacy, Clinical notes de-identification, Homomorphic encryption

## Abstract

**Background:**

Medical data sharing is a big challenge in biomedicine, which often hinders collaborative research. Due to privacy concerns, clinical notes cannot be directly shared. A lot of efforts have been dedicated to de-identifying clinical notes but it is still very challenging to accurately locate and scrub all sensitive elements from notes in an automatic manner. An alternative approach is to remove sentences that might contain sensitive terms related to personal information.

**Methods:**

A previous study introduced a frequency-based filtering approach that removes sentences containing low frequency bigrams to improve the privacy protection without significantly decreasing the utility. Our work extends this method to consider clinical notes from distributed sources with security and privacy considerations. We developed a novel secure protocol based on private set intersection and secure thresholding to identify uncommon and low-frequency terms, which can be used to guide sentence filtering.

**Results:**

As the computational cost of our proposed framework mostly depends on the cardinality of the intersection of the sets and the number of data owners, we evaluated the framework in terms of these two factors. Experimental results demonstrate that our proposed method is scalable in various experimental settings. In addition, we evaluated our framework in terms of data utility. This evaluation shows that the proposed method is able to retain enough information for data analysis.

**Conclusion:**

This work demonstrates the feasibility of using homomorphic encryption to develop a secure and efficient multi-party protocol.

## Background

Clinical notes represent an indispensable component of electronic health records (EHRs), which contain important information (such as symptoms and medical history) that structured data might not cover. Sharing clinical notes can promote research, improve healthcare services, and contribute to clinical decision support [1]. However, it has been a very challenging task to de-identify such data to mitigate the privacy risks. Due to the unstructured nature of notes, de-identification is not as straightforward as for the structured data. To satisfy the privacy regulations of Health Insurance Portability and Accountability Act (HIPAA), we can remove the Protected Health Information (PHI) defined in the HIPAA safe harbor method. Traditionally, this is done through the detection and scrubbing of 18 specific categories of PHIs including name, social security number, dates, etc. Many efforts have been devoted in this direction including both the manual and the automatic approaches. Manual approaches to identify PHI are prone to mistakes (Neamatullah et al [2] shows the recall of 14 clinicians to detect 130 clinical notes varied from 0.63 to 0.94) and they are also expensive (e.g., ~$50/h to read and label 20 k words/hour in de-identifying MIMIC II database [3]). Automated algorithms can save time and reduce the human review efforts. Early systems used rule or template based approaches to match and detect PHI [4]. Berman [5] developed a concept matching algorithm that steps through confidential pathology text to replace medical terms matching standard nomenclature code with a synonymous term while keeping the high frequency “stop words” intact. However, the system blocks too much and has a high false positive rate, making the outputs hard to read [2]. Finley et al proposed a similar method which was applied to de-identify distributed semantic models [6]. Scrub system [7] used a template-based approach to match components of high privacy risk, which are then removed, generalized, or replaced with made-up ones. This method can get rid of explicit personally-identifiable information but it does not handle combinations of fields and the results might still be matched or linked to the identities of individuals [8].

Other researchers also treated text de-identification as a classic Named Entity Recognition (NER) problem and tried to solve it with machine learning models [9]. Szarvas et al used decision tree to take into consideration of various features (length, frequency, etc.) to detect PHIs [10]. Several research groups [2, 11] developed methods based on Support Vector Machine (SVM) to classify sensitive attributes based on Part-of-speech (POS) inputs. Another popular framework utilizes conditional random fields (CRF), an extension of logistic regression and considers correlations in the sentence to predict PHIs [12, 13]. Latest methods in this direction [14] using deep learning approaches reported improved performance in detecting PHIs but the model requires careful tuning of parameters for each dataset, which makes it hard to be portable for collaborative research.

A recent method was proposed by Li et al [15] to filter out rare sentences (frequency < 3) and sentences containing bigrams under a certain frequency threshold (frequency < 256). This method demonstrated good performance in obtaining sentences with almost no PHIs (evaluated by a manual review on sampled outputs) while preserving a similar *Type Unique Identity* (TUI) distribution of the original data, providing an alternative and generalizable way to obtain useful data with mitigated privacy risks. However, the method is only designed to anonymize data from a single source. In reality, collaborative research often involves more than one party and poses new challenges to conduct filtering in a global manner. In this paper, we propose a distributed and privacy-preserving method as an extension of the single source model [15]. Our criterion for bigram filtering is stricter than previous work [15] by taking distributional differences of local sites into consideration. We will only keep sentences containing bigrams observed at all collaboration sites and with sufficient global frequency. Our proposed method can be easily generalized to cover other NLP artifacts including unigram, trigram, and n-gram. To develop such a global bigram-based filtering method, appropriate protection needs to be enforced on private set intersection, secure count aggregation, and thresholding to ensure data confidentiality during the process.

### Existing works and their limitations

A critical step for our distributed bigram filtering model is to find what the bigrams in common are among all collaborative sites in a privacy-preserving manner. Although there are several studies on 2-party private set intersection [16, 17], only a few works have been done to solve multi-party private set intersection (MPSI) problem. Earlier approaches for MPSI have some limitations. In [18], the dataset size of each party must be equal. Another approach suffers from approximation errors [19]. A recent work has shown the feasibility of handling *n > 2* parties [20]. In this work [20], each data owner constructs a Bloom filter from their data (using only the words or bigrams, not the count associated with them). Data owners send the encrypted (exponential ElGamal encryption scheme) Bloom filter to a service provider. All encrypted Bloom filters are securely added by the service provider without decrypting, which results in an encrypted Integrated Bloom Filter (IBF). Then, the service provider constructs a randomized *n*-subtraction of IBF (encrypted), where *n* is the number of parties. The service provider broadcasts this encrypted randomized *n*-subtraction of IBF to all the data owners. Finally, all data owners jointly decrypt it and compute the set intersection: if an element *x* is in the set intersection, the corresponding array locations in the encrypted randomized *n*-subtraction of IBF, where *x* is mapped by *k* hash functions is an encryption of 0; otherwise, is an encryption of random integer. Their approach [20] demonstrated good performance for set sizes range from 64 to 16,384. However, this approach may not scale well with millions of records, which is common in real world applications. With a much larger set, to reduce the probability of false positives, the size of the Bloom filter should be large enough compared to the number of items to be inserted into it. In their approach, runtime is dominated by the encryption and decryption of Bloom filter. Constructing, encrypting, and transferring such large Bloom filters (that can deal with millions of records with a minimal probability of false positives) will introduce huge computation and communication overhead.

Our problem specification is different from these works on private set intersection mentioned here, which do not involve any secure thresholding operations. We are describing these works just to give an overview of state-of-the-art solutions of the related problems. To the best of our knowledge, there is no secure protocol for sensitive information filtering that combines private set intersection and secure thresholding.

The major contributions of this article are summarized as follows:

1. We propose a novel framework based on private set intersection and secure thresholding to identify uncommon and low-frequency bigrams, which can be used to remove sentences from clinical notes that might contain privacy sensitive terms. The proposed framework takes into consideration distributional differences of local sites. In addition, the framework is highly generalizable: it can be used for any other type of NLP artifact.
2. The proposed framework demonstrates the feasibility of using homomorphic encryption to develop a secure and efficient multi-party protocol. For the homomorphic operations, we leverage a Single Instruction, Multiple Data (SIMD) scheme that significantly boosts the performance of the proposed framework.
3. Our proposed method can simultaneously guarantee data privacy and preserve data utility for analysis. It is able to retain enough information for data analysis.

To the best of our knowledge, this is the first privacy-preserving work to de-identify clinical notes from distributed sources.

## Implementation

### System overview

We developed a secure and privacy-preserving framework for bigram-based filtering to simultaneously meet two goals: *multiparty private set intersection* and *secure thresholding*.

#### Architecture and entities

**Fig. 1:**
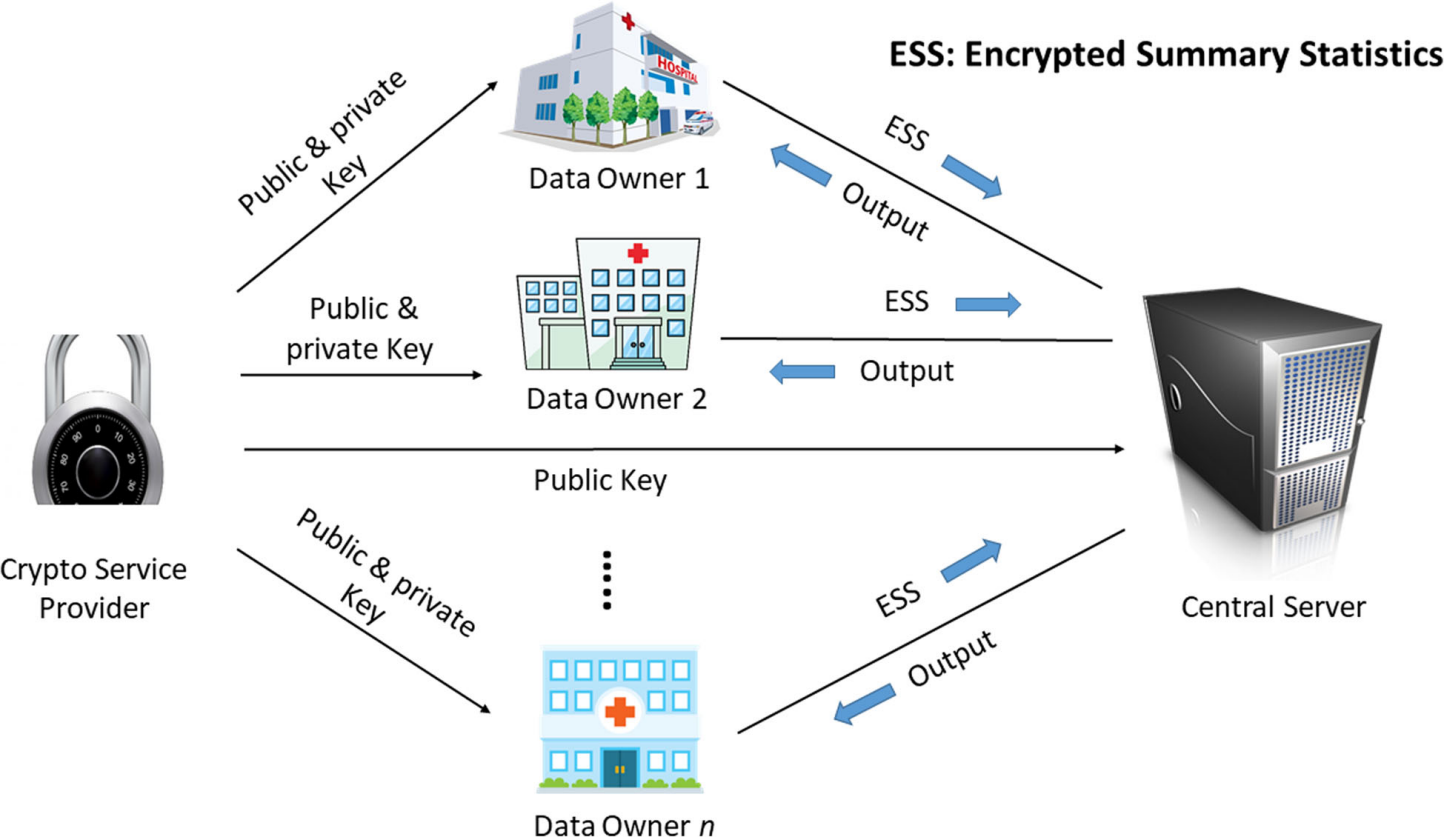
Block diagram of the system architecture. Only encrypted summary statistics are delegated to the central server to conduct the bigram filtering, which returns to individual data owners with encrypted bigrams (that are both common and frequent enough in a global manner). This block diagram was drawn by the authors

There are three types of entities in our system. Figure 1 represents the system architecture of our proposed framework.

- *Data owner*: Data owners might be any hospital, clinical research facility, or federal (or, provincial) health science institute that possess clinical datasets. Our proposed system supports any number of data owners.
- *Crypto Service Provider (CSP)*: Cryptographic Service provider manages public and private keys. CSP also manages salt for hashing (refer to Security Analysis, Security of Hashing for more details). Each data owner receives a public key, a private key, and an evaluation key from the CSP. Data owners use public key to encrypt their data (count of bigram), and use private key to decrypt the encrypted response from the central server.
- *Central Server*: The central server coordinates the system protocol. It maintains communications with all other entities of the system. It receives encrypted data (hash and encrypted count of bigram) from the data owners, performs computations locally, and finally sends the encrypted result to the data owners.

#### Threat model

In this work, our goal is to ensure that each data owner knows the thresholded set intersection as a result of the protocol. Data owners should not know the elements of other data owners’ dataset (elements that are not in the intersection). We consider the central server as a semi-honest party (also known as honest-but-curious). It follows the protocol but may attempt to scoop additional information from the server logs or received messages. We also assume that the data owners do not collude. These assumptions are standard and have been adopted by several earlier works [21, 22].

### Problem specification

The objective of this study is to identify the globally infrequent common bigrams of participating parties based on a threshold value. In the first phase of the system protocol, all the parties jointly identify the common bigrams. Then, data owners send counts of the common bigrams to the central server. Consider the example of Table 1. Here, data owner *A* sends *E* (count of bigram Flu-fever = 10), *E* (count of bigram Cancer-pain = 15), and *E* (count of bigram Diabetes-glaucoma = 20), where *E* denotes an encryption algorithm. After receiving counts from all data owners, the central server performs addition over the bigram counts. If the total count for a specific bigram is less than a predetermined threshold, then that bigram is considered privacy-sensitive, and this information can be used to guide sentence filtering of clinical notes. The intuition behind this filtering is: the more potentially identifying a bigram is, the rarer it will be.

**Table 1:** Identification of globally infrequent bigrams

Data Owner	Frequency of the bigram Flu-fever	Frequency of the bigram Cancer-pain	Frequency of the bigram Diabetes-glaucoma
A	10	15	20
B	20	15	10
C	5	15	25
Total	35	45	55

Let us consider the data of the above table. Assume, the threshold value is 40. Since total count of Flu-fever (35) is less than the threshold value (40), it will not be considered privacy-sensitive

### Preliminaries

#### Homomorphic encryption

The concept of an encryption scheme that can perform arbitrary computation on encrypted data was first proposed by Rivest et al [23] in 1978. Many traditional homomorphic encryption schemes are either additively homomorphic (Paillier [24]), or multiplicatively homomorphic (ElGamal [25]). However, such restriction to one single algebraic operation is very inconvenient for general purpose applications. Lately, researchers are adopting lattice cryptosystems, which leverage ring homomorphism (addition and multiplication) [26, 27]. The cryptosystem in [28] is a Somewhat Homomorphic Encryption (SWHE) scheme that can compute a bounded number of homomorphic functions. Other recent RLWE-based SWHE cryptosystems include BGV [29], FV [30], and YASHE [31]. While these systems are intrinsically similar, there are differences and trade-offs. Interested readers can refer to [32] for more details.

In this work, we used the FV cryptosystem (other RLWE-based system will work in a similar manner), which consists of the following functionalities:

- *KeyGen (params)*: Given the system parameters *params* as input, *Keygen* generates a public-private key pair and an evaluation key *(pk, sk, evk)*.
- *Enc (pk, m)*: An encryption algorithm encrypts a plaintext message *m* using the public key *pk*.
- *Dec (sk, c):* Let, *c* be the encryption of a plaintext *m.* A decryption algorithm outputs *m,* given private key *sk* and ciphertext *c* as input.
- *Add(c*_1_, *c*_2_*)*: Let *c*_1_, *c*_2_ be the ciphertexts for messages *m*_1_, *m*_2_ respectively. Given, *c*_1_, *c*_2_ as input, a homomorphic addition operation *Add* computes the encrypted sum of *m*_1_, *m*_2_*.*
- *Mult(c*_1_, *c*_2_*)*: Let *c*_1_, *c*_2_ be the ciphertexts for messages *m*_1_, *m*_2_ respectively. Given, *c*_1_, *c*_2_ as input, a homomorphic multiplication operation *Mult* computes the encrypted product of *m*_1_, *m*_2_*.*
- *ReLin(c*_*mult*_, *evk):* The objective of relinearization operation *ReLin* is to reduce the size of a given ciphertext *c*_*mult*_ back to (at least) 2. Relinearization is performed when the size of the ciphertext increases substantially by multiplication operations. Relinearization operation requires the evaluation key *evk*.

There is a recent application of homomorphic encryption, which can securely perform genome search on a semi-honest cloud server [33].

#### Ciphertext packing

The considerable computational overhead of homomorphic encryption results from the large ciphertexts. As homomorphic operations have to operate on these large ciphertexts, they can be quite slow. The primary solution to deal with this issue is to work with packed ciphertexts, which refer to the ciphertexts that encrypt a vector of plaintext values [34, 35]. Homomorphic operations can be performed on these vectors component-wise in a Single Instruction, Multiple Data (SIMD) manner. Depending on the memory allowance, this mechanism can significantly boost the performance due to parallelization.

Consider the plaintext elements in a polynomial quotient ring *m* ∈ *R*_*t*_ = *Z*_*t*_/(*X*^*n*^ + 1) and ciphertext elements in *R*_*q*_ = *Z*_*q*_/(*X*^*n*^ + 1). Here, *q* and *t* are positive integers (*q* > *t*, *q* > 1, see [30]), *Z*_*q*_ represents the set of integers $\left(-\frac{q}{2},\frac{q}{2}\right]$, and *X*^*n*^ + 1 is an irreducible polynomial of degree *n*. Using ciphertext packing, we can encrypt *n* plaintext values in a single ciphertext for a single instruction execution.

Since a packed ciphertext is essentially the same as a standard ciphertext, the basic homomorphic operations still work, for instance, homomorphic addition by adding ciphertexts. Ciphertext packing thus facilitates SIMD-type homomorphic computation, which is capable of computing the same function over many inputs at once. The usage of ciphertext packing in our proposed framework is elaborated in Detailed System Protocol.

We apply ciphertext packing to minimize both computational and communication overhead. The data owners group their counts of bigrams into vectors of length *n*, encrypt them, and send *Cardinality of Intersection of Sets/ n* ciphertexts to the central server (see Detailed System Protocol). Then the packing mechanism allows the central server to perform computation on *n* items simultaneously, which results in *n*-fold improvement in computation and communication both. In our case, *n* equals to 4096, which leads to a significant time cost reduction over the naive homomorphic encryption method.

#### Hash functions

Hash functions are one of the fundamental cryptographic primitives. Hash functions can compute a digest of a given message, which is a fixed-length bit string. For a given message, the message digest (also known as hash value or hash) can be considered as an unique representation of that message. In this work, we have used SHA-256, which is a member of Secure Hash Algorithm (SHA) family. The length of message digest for SHA-256 is 256 bits [36]. Security of hashing is discussed in detail in Security Analysis, Security of Hashing.

### Detailed system protocol

**Fig. 2:**
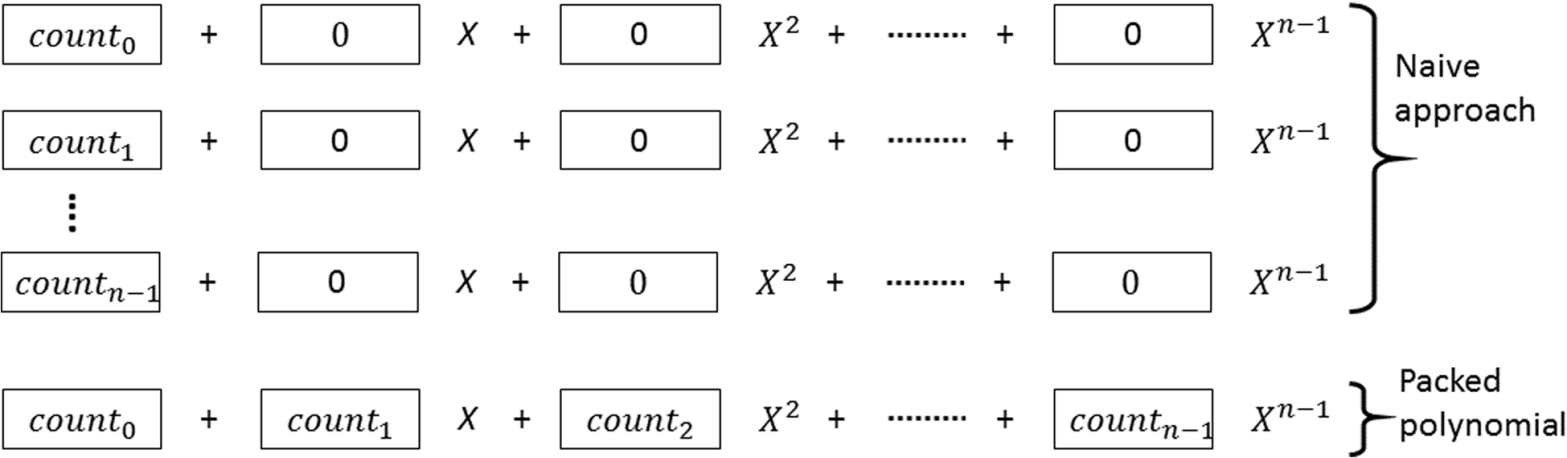
Flow diagram for the proposed system protocol. The order of the execution runs in a top down manner in key distribution and computation phases

At the system initialization phase, data owners receive public and private keys from the CSP. Also, the central server receives only the public key. Then, each data owner sends the hashes of bigrams to the central server. After receiving the hashes from each data owner, the central server computes the intersection of the hashes. Then, the central server sends the elements of this intersection to data owners. Figure 2 shows the flow diagram of our protocol.

**Fig. 3:**
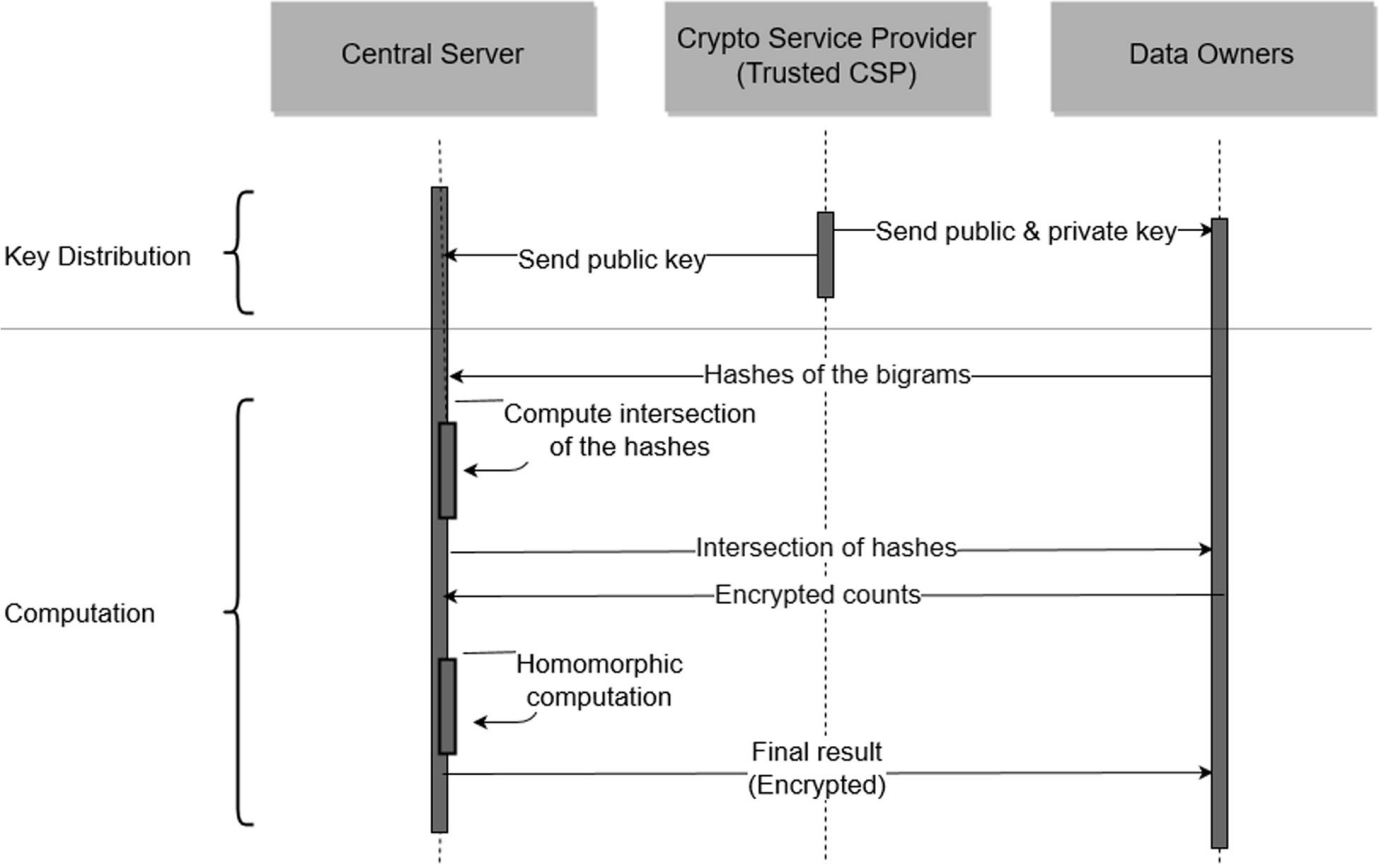
Usage of ciphertext packing in our proposed method. Here, *n* is the degree of the polynomial, which indicates the number of slots for parallel computing

Upon receiving the intersection of the hashes from the central server, data owners encrypt the local frequency of the intersected bigrams by using the ciphertext packing technique. To do so, they follow the order received from the central server. Figure 3 illustrates this technique for a data owner and indicates the difference with naive homomorphic encryption approach. After encrypting the counts, data owners send the packed ciphertexts to the central server, where the encrypted global frequency will be computed.

After receiving the ciphertexts, the central server performs homomorphic addition operation on these packed ciphertexts. So, at the end of this addition process, the resulting output looks like the table below. Here, *E* represents the encryption function.

In Table 2, *E*(C11) denotes the encrypted count of bigram B1 contributed by data owner 1. *E*(C12) denotes the encrypted count of B1 contributed by data owner 2, *E*(C13) denotes the encrypted count of B1 contributed by data owner 3, and so on.

**Table 2:** Secure count aggregation at central server

Bigram	Encrypted Global Frequency
B1	*E*(C11) + *E*(C12) + *E*(C13) + ⋯
B2	*E*(C21) + *E*(C22) + *E*(C23) + ⋯
B3	*E*(C31) + *E*(C32) + *E*(C33) + ⋯
⋮	⋮

Now, we need to meet the thresholding requirement for the sum of homomorphically encrypted counts. For each of the records, we check the following inequality.

$$ E(C11)+E(C12)+E(C13)+\cdots >\mathrm{threshold} $$

Solving this problem involves both addition and comparison. It is known that in arithmetic circuits, addition is cheap but comparison is not trivial. To avoid the comparison operation in the arithmetic circuit, we formulate the problem in the following way,

$$ E(C11)+E(C12)+E(C13)+\cdots - threshold $$

After performing the above mentioned homomorphic operation, the central server sends to the data owners r*(*E*(*C*11) + *E*(*C*12) + *E*(*C*13) + ⋯ − *threshold*), where *r* is a random number drew by the central server. After decrypting it, if a data owner gets a random negative number (or zero), she will understand that the sum of counts of the corresponding record is less than (or equal to) the threshold. Similarly, if a data owner gets a random positive number, she will understand that the sum of counts of the corresponding record is greater than the threshold. Multiplying every coefficient of the resulting ciphertext by same random number may expose some additional information about other data owners’ counts. So, we multiply the resulting ciphertext with a random polynomial, all of whose coefficients are randomly generated.

Although polynomial addition and subtraction are coefficient-wise by nature, polynomial multiplication in *R*_*t*_ (and *R*_*q*_) is a convolution product of the coefficients. An effective technique to transform convolution product into coefficient-wise product in polynomial ring is the Number-Theoretic Transform (NTT), a specialization of Fourier transform for finite rings. One important property of NTT is that it works in the same ring as lattice cryptosystems do. Therefore, NTT can be used to improve the efficiency of the polynomial operations [37]. To ensure that the products in the ciphertext space be translated into coefficient-wise products in plaintext space, we perform an inverse-NTT operation to plaintext before encryption and a NTT operation after decryption.

## Results

### Experimental settings

#### Dataset

We used the MIMIC-III (Medical Information Mart for Intensive Care), an openly available dataset comprising of de-identified health data associated with ~ 40 k critical care patients [38]. To be specific, we used NOTEEVENTS table of this database, which contains de-identified clinical notes including nursing and physician notes, and reports on ECG, radiology, and discharge summary. There are 2,083,180 rows in NOTEEVENTS table.

#### Dataset preprocessing

The *text* column of NOTEEVENTS table represents the contents of the clinical notes. At first, we removed the stop words from the entries of this column. We also removed any standalone symbol/character, numerical values including temporal expressions (e.g., 4:10 AM, 9:50 PM).

#### Evaluation environment

Experiments were performed on Google Compute Engine (GCE) and Amazon EC2 cloud server. GCE is a cloud computing service that provides virtual machines running in Google’s data centers.

In GCE, we used a *n1-standard-8* machine with Ubuntu 16.04.3 LTS. For Amazon EC2, the configuration was *r3.xlarge* with Ubuntu 16.04.2 LTS. The central server was hosted in Amazon EC2 and the CSP and the data owners were hosted in GCE. Each entity of the system architecture communicated with others through TCP (Transmission Control Protocol).

#### Implementation

To hash the words, SHA-256 (OpenSSL version 1.0.2 g) was used. To encrypt the bigram counts, we use FV scheme [30]. For FV implementation, we choose NFLlib [39]. NFLlib [39] is an efficient and scalable C++ library for ideal lattice cryptography. In our implementation, the computation and communication tasks are processed in parallel whenever possible. We used OpenMP for this purpose. An open-source implementation of our proposed framework is available at GitHub.

### Experimental results

It is evident from the description of our proposed method that the runtime mostly depends on the cardinality of the intersection of the sets and the number of data owners. We evaluated our proposed method in terms of these two factors. Tables 3 and 4 show the experimental results. These tables report computation time for intersecting hashes, encryption, homomorphic operation, decryption, and network communication costs. However, the total time reported here does not include cost for system initialization, for instance, reading and parsing configuration file, reading input data file, TCP socket setup and shutdown etc.

**Table 3:** Experimental results for different cardinality of intersection of sets. In the five different settings, cardinality is increased by 1% of the entire dataset. The number of data owners is a constant [3]. The numbers are in seconds

Cardinality of Intersection	Intersecting Hashes (s)	Encryption (s)	Homomorphic Operation (s)	Decryption (s)	Network Comm. (s)	Total Time (s)
1,515,520 (~ 10%)	4.63	8.11	55.43	6.73	0.48	75.38
1,667,072 (~ 11%)	4.69	8.92	61.19	7.06	0.52	82.38
1,818,624 (~ 12%)	4.98	9.70	66.63	7.88	0.54	89.73
1,970,176 (~ 13%)	5.07	10.97	72.21	8.49	0.59	97.33
2,121,728 (~ 14%)	5.20	11.32	77.65	9.34	0.60	104.11

**Table 4:** Experimental results for different number of data owners. The cardinality of intersection of sets is fixed, which is 1,515,520. The numbers are in term of seconds

Number of Data Owners	Intersecting Hashes (s)	Encryption (s)	Homomorphic Operation (s)	Decryption (s)	Network Comm. (s)	Total Time (s)
2	1.69	8.17	54.72	6.29	0.32	71.19
3	2.72	8.19	55.49	6.33	0.39	73.12
4	3.53	8.28	55.51	6.60	0.46	74.38
5	4.63	8.22	56.36	6.67	0.53	76.41
6	5.36	8.24	58.01	7.11	0.60	79.32

#### Communication cost

The total number of bigrams was about 15 million. These were equally distributed among three data owners for the experiments shown in Table 2. Each data owner was given 4 million bigrams along with common ones as shown in the first column of Table 2. For five different settings, the sizes of encrypted data for each data owner were 46.3, 51, 55.6, 60.2, and 64.8 MB respectively. The sizes of the files containing hashes (for each data owner) were 341, 351, 360, 370, and 379 MB respectively. For the experiments shown in Table 3, bigrams were distributed equally among the six data owners (3,518,464 each). The size of the encrypted data for each data owner was 46.3 MB. The size of the file containing hash (for each data owner) was 218 MB.

## Discussion

### Concept distribution analysis

Now, we show that the proposed method is able to retain enough information for data analysis. We compare the concept distribution of clinical notes and sanitized sentence repository constructed by eliminating sentences of the clinical notes that contain low frequency bigrams (frequency less than or equal to a specified threshold). Due to the significant computations involved, we sampled 800 clinical notes for this experimentation. The results of concept distribution analysis are reported in Table 5. Each concept is expressed as a *Type Unique Identity* (TUI) defined by UMLS [40]. The difference of the TUI distribution is not too large when the threshold is small but it gets larger at an increasing threshold. However, this is not a critical issue because we can maintain the original distribution by oversampling the filtered corpus using sentences that contain one or more TUIs. This is a standard combinatorial optimization problem but we do not explore it in this paper.

**Table 5:** Comparison of TUI Proportion Distribution

TUI	Original Clinical Note	Threshold = 1	Threshold = 2	Threshold = 4	Threshold = 8	Threshold = 16
T007	0.2627	0.2012	0.1601	0.1421	0.0922	0.0428
T023	5.8168	4.4492	3.5281	2.9490	2.5213	2.1758
T033	7.7646	5.3959	4.8470	3.6402	3.1259	2.5570
T047	7.6978	5.4338	4.8742	3.7598	3.3876	2.8825
T060	2.5509	1.8672	1.6446	1.4018	1.1242	0.9680
T074	1.5871	1.2046	1.0991	0.9302	0.8257	0.6724
T093	0.9824	0.7123	0.6594	0.5846	0.5197	0.4925
T109	4.1908	2.8163	2.7084	2.8069	2.6024	1.6447
T121	1.2840	0.8898	0.8983	0.7719	0.5971	0.6253
T170	0.7523	0.5182	0.4450	0.3165	0.2764	0.1284
T184	3.5566	2.4968	2.2498	1.8443	1.4265	0.6895
T201	1.8249	1.1075	0.9960	0.9173	0.8441	0.8437

### Security analysis

In this section, we analyze the security of our proposed framework.

#### Security of encryption

To evaluate the security of a lattice cryptosystem, a widely used measure is root-Hermite factor . Lindner and Peikert showed a mathematical relationship between root-Hermite factor and security level *λ* (in bits) [41].



 is given by,  where $c\approx \sqrt{\frac{\mathit{\ln}\left(1/\epsilon \right)}{\varPi }}$ and $s=\sigma \sqrt{2\varPi }$.

*n*, *q*, *and s* represent the degree of the polynomial ring, ciphertext modulus, and scale parameter of the error distribution respectively. *σ* denotes the standard deviation of the error distribution, and *ϵ* is the attacker advantage.

For our experiments, we choose *n* = 2^12^, *q* = 2^120^, *σ* = 3, *ϵ* = 2^−32^. According to root-Hermit factor measure, our proposed method guarantees 142 bit security.

#### Security of hashing

One of the primary security requirements of hash function is one-wayness: given a hash output *h*, it must be computationally infeasible to find an input *m* such that *h* = *H*(*m*). In other words, given a message digest, an adversarial cannot find out the matching message *m* from *H*^−1^(*h*) = *m*. There exist some cryptanalytic attacks against one-way hashing that try to break the security properties of the hash function. Brute-force attack (also known as exhaustive search) is a type of cryptanalytic attack. Let (*m*, *h*) denote the pair of input message and output hash value, and let *M* = {*m*_1_, *m*_2_, .. …, *m*_*k*_} be the message space of all possible messages *m*_*i*_. Such an attack checks for every element of *M* if *H*(*m*_*i*_) =  = *h*. If an equality holds, a possible input message is found. This type of attack is impractical for a large message space. A similar one is called dictionary attack, which tries all the input messages in a pre-arranged listing, generally derived from a list of words such as in a dictionary (hence the term *dictionary attack*), which has a smaller space to search. There is a variant of dictionary attack, known as Rainbow table attack [42], which uses a precomputed table (rainbow Table [42] that contains elements up to a certain length consisting of a limited set of characters) for reversing hash functions. This attack requires less computation time but more storage compared to brute-force attack. Addressing above mentioned attacks, we used salt to randomize the hashing. In cryptography, salt refers to random data that are used as an additional input to a hash function. Salt was generated by the CSP and provided to data owners before each hashing process, making these attacks computationally infeasible.

Another desirable property of a hash function is collision resistance. A hash function is said to be collision resistant if it is computationally infeasible to find two different inputs *m*_1_ ≠ *m*_2_ with *H*(*m*_1_) =  = *H*(*m*_2_). It seems if the hash function has an output length of *b* bits, we have to check about 2^*b*^ messages. However, it turns out that an attacker needs only about 2^*b*/2^ messages. This is a quite surprising result, which is due to the birthday attack. This attack is based on the birthday paradox, which is a powerful tool that is often used in cryptanalysis.

Collision search for a hash function *H*() is exactly the same problem as finding birthday collisions among party attendees: how many people are required at a birthday party such that there is a significant chance that at least two attendees have the same date of birth?. The question is how many messages (*m*_1_, *m*_2_, ……, *m*_*k*_) does an attacker need to hash until he has a chance of finding *H*(*m*_*i*_) =  = *H*(*m*_*j*_) for some *m*_*i*_ and *m*_*j*_ that he chooses. The most significant consequence of the birthday attack is that the number of messages needed to hash to find a collision is approximately equal to the square root of the number of possible output values, i.e., about $\sqrt{2^b} = {2}^{b/2}$. Hence, for a security level of *u* bit, the hash function needs to have an output length of 2*u* bit. In order to prevent collision attacks based on the birthday paradox, the output length of a hash function must be at least 128 [36]. As mentioned previously, we are using SHA-256 in this work, which has output length 256.

In 2004, collision-finding attacks against MD5 and SHA-0 were demonstrated by Xiaoyun Wang [43]. One year later, it was claimed that the attack could be extended to SHA-1 and a collision search would take 2^63^ steps, which is considerably less than the 2^80^, achieved by the birthday attack (the output width in this case is 160 bit). In this work, we are using SHA-2 (precisely, SHA-256) against which no attacks are known to date.

## Conclusion

In this article, we proposed a novel protocol to achieve the joint mission of private set intersection and secure thresholding for a distributed data de-identification task. We extended a previous filtering-based method to cover data from distributed sources and demonstrated the feasibility of using homomorphic encryption to develop an efficient multi-party protocol for distributed data de-identification. Experimental results show that our proposed method can simultaneously guarantee data privacy and preserve data utility for analysis.

To the best of our knowledge, this is one of the pioneering privacy-preserving initiatives to de-identify clinical notes in a distributed environment. We have open sourced our code in GitHub with a GNU general public license, along with a software manual for compiling and running it.

### Availability and requirements

Project name: A Privacy-preserving Distributed Filtering Framework for NLP Artifacts.

Project home page: https://github.com/Nazmus-Sadat/th_mpsi

Operating system: Linux.

Programming language: C++.

License: GNU general public license.

## Data Availability

The clinical notes used in the experiment are available from MIMIC-III (Medical Information Mart for Intensive Care), an openly available dataset [38].

## Abbreviations

CSP: Crypto Service Provider
EHR: Electronic Health Record
GCE: Google Compute Engine
HIPAA: Health Insurance Portability and Accountability Act
IBF: Integrated Bloom Filter
MIMIC-III: Medical Information Mart for Intensive Care
MPSI: Multi-party private set intersection
NER: Named Entity Recognition
NTT: Number-Theoretic Transform
PHI: Protected Health Information
SHA: Secure Hash Algorithm
SIMD: Single Instruction, Multiple Data
SWHE: Somewhat Homomorphic Encryption
TCP: Transmission Control Protocol
TUI: Type Unique Identity
UMLS: Unified Medical Language System
